# HFD-induced hepatic lipid accumulation and inflammation are decreased in Factor D deficient mouse

**DOI:** 10.1038/s41598-020-74617-5

**Published:** 2020-10-16

**Authors:** Hiromi Tsuru, Mizuko Osaka, Yuichi Hiraoka, Masayuki Yoshida

**Affiliations:** 1 Department of Life Sciences and Bioethics, Graduate School of Medical and Dental Sciences, Tokyo Medical and Dental University (TMDU), 1-5-45, Yushima, Bunkyo-ku, Tokyo, 113-8510 Japan; 2 Department of Nutrition and Metabolism in Cardiovascular Disease, Graduate School of Medical and Dental Sciences, Tokyo Medical and Dental University (TMDU), Tokyo, Japan; 3 Laboratory of Molecular Neuroscience, Medical Research Institute, Tokyo Medical and Dental University (TMDU), Tokyo, Japan

**Keywords:** Diseases, Endocrinology

## Abstract

Excessive intake of fat causes accumulation of fat in liver, leading to non-alcoholic fatty liver disease (NAFLD). High-fat diet (HFD) upregulates the expression of Factor D, a complement pathway component, in the liver of mice. However, the functions of Factor D in liver are not well known. Therefore, the current study investigated the relationship between Factor D and hepatic lipid accumulation using CRISPR/Cas9-mediated Factor D knockout (FD-KO) mice. Factor D deficiency downregulated expression of genes related to fatty acid uptake and de novo lipogenesis in the liver. Furthermore, Factor D deficiency reduced the expression of inflammatory factors (*Tnf* and *Ccl2*) and fibrosis markers and decreased accumulation of F4/80-positive macrophages. These data suggest that the Factor D deficiency improved hepatic lipid accumulation and hepatic inflammation in HFD-fed mice.

## Introduction

Non-alcoholic fatty liver disease (NAFLD) is characterized by excessive hepatic fat accumulation via lipid and/or carbohydrate intake^1^. Metabolic disorders including dyslipidaemia, obesity, and type 2 diabetes often comorbid with NAFLD^2^. In fact, increased occurrence of NAFLD has paralleled recent increases in the prevalence of obesity and diabetes^3^. Clinically, NAFLD shares similar risk factors with cardiovascular disease and is associated with the development of hepatocellular carcinomas^4,5^ . NAFLD livers exhibit an infiltration of inflammatory macrophages and other myeloid cells in the hepatic parenchymal area, implicating these mechanisms in the pathogenesis of NAFLD^6^. Hepatic lipid elevation and inflammation has also been associated with NAFLD^7^, however, the precise mechanism by which hepatic lipid accumulation and inflammation contribute to NAFLD remains poorly understood.

Liver is not only the central organ for lipid metabolism, but also serves as the main source of several key inflammatory cascade molecules, including complement factors. Previous clinical studies implicated complement factors in NAFLD. Deposition of activated C3 and C4d has been shown in the liver of NAFLD patients^8^. Additionally, serum C3 levels have been correlated with NAFLD prevalence and hepatic lipid accumulation^9,10^. We therefore hypothesized that complement factors may play a role in fat intake and accumulation in the liver. Recent studies demonstrated that mRNA levels of Factor D, a complement system component, increased dramatically in livers of mice fed high-fat diet (HFD)^11,12^. Factor D or adipsin, a serine protease in the alternative complement pathway, cleaves Factor B to form two fragments: Ba and Bb^13^. Factor D is synthesized in liver and adipocytes where it regulates cell differentiation and lipid accumulation^14,15^. Factor D has also been shown to improve insulin secretion in diabetic mice^16^. Furthermore, ethanol-induced liver injury has been reported to be more severe in the absence of Factor D^17^. Based on these previous studies, we investigated the role of Factor D in the development of NAFLD and hepatic lipid accumulation in mice.

## Results

### HFD dramatically increased hepatic lipid accumulation in wild-type mice

**Figure 1:**
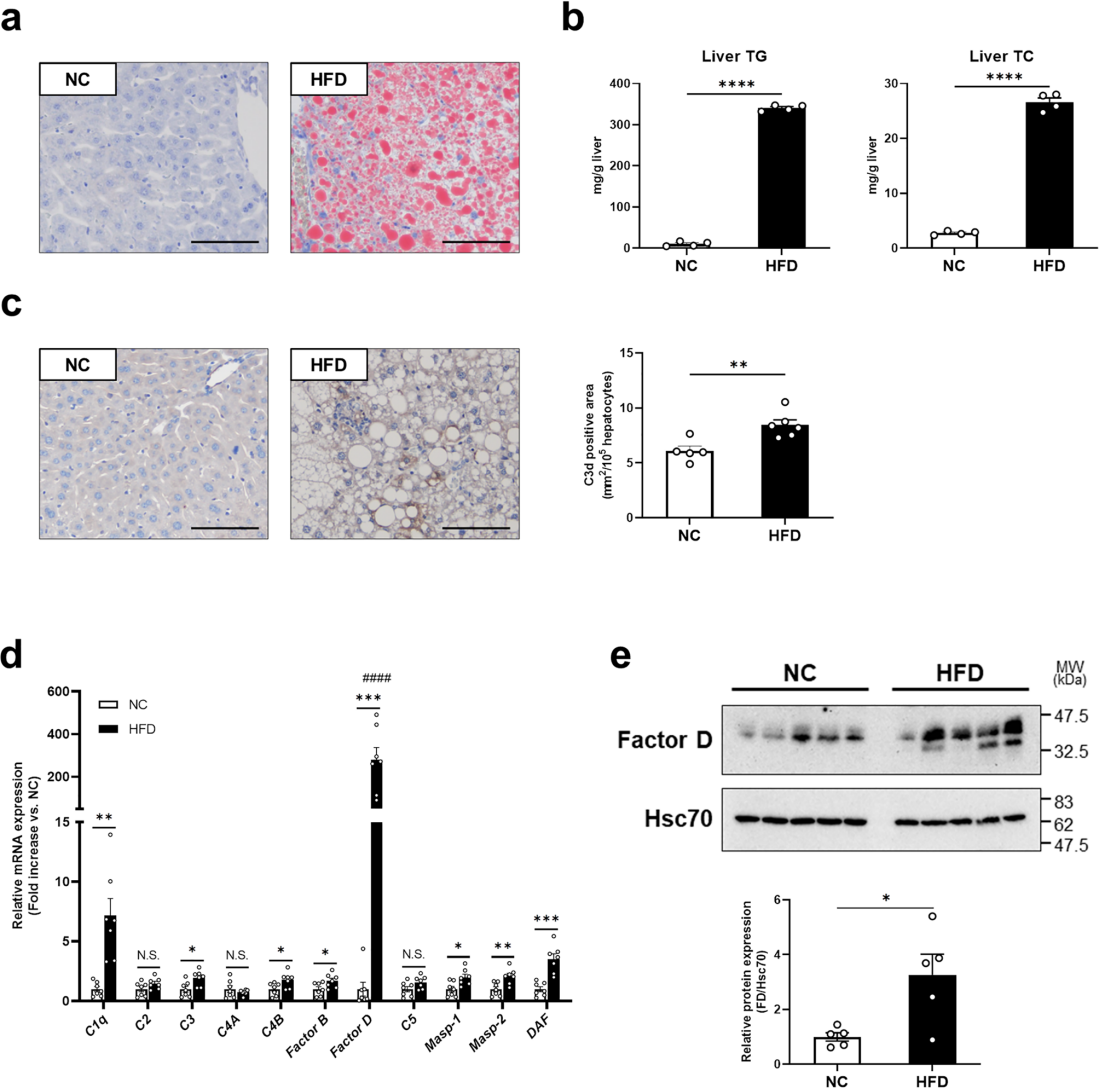
HFD for 17 weeks remarkedly increased hepatic lipid accumulation and Factor D expression in WT mice.** (a**) Images of Oil Red O staining for sections of livers. HFD-feeding enhanced lipid accumulation. Bars, 100 μm. Magnification,  × 20 objective. (**b)** TG or TC levels in liver. TG or TC level in liver increased by HFD. N = 4 per group. ****p < 0.0001 by an unpaired two-sided *t* test. (**c**) Images of immunohistochemistry for C3d in liver (left panels). Bars, 100 μm. Magnification,  × 20 objective. (**c**) Right graph showed quantification of C3d-positive area. HFD increased C3d deposition in mice. N = 5, 6. *p < 0.01 by an unpaired two-tailed *t* test. (**d**) Relative mRNA expression levels of complement components in liver of WT mice fed NC or HFD. N = 7 in each group. *p < 0.05, **p < 0.01, ***p < 0.001 by an unpaired two-tailed *t* test. #### *p* < 0.0001 were calculated using one-way ANOVA with Dunnett's multiple comparisons test. (**e**) Representative images of western blotting for Factor D in liver (upper panels) and graph for relative Factor D expression levels normalized to Hsc70 (lower panel). Specific bands disappeared in FD-KO mice (Supplementary Fig. 1c, d). These images were cropped and the full images were shown in Supplementary Fig. 1. N = 5 in each group. *p < 0.05 by an unpaired two-tailed *t* test. Data are shown as mean ± SEM.

In wild-type (WT) mice, 17 weeks of HFD resulted in significantly higher liver fat accumulation compared with mice fed normal chow (NC). Oil Red O staining of liver sections from HFD-fed mice showed a significant increase in neutral lipid droplets (Fig. 1a) with elevated hepatic triglyceride (TG) and total cholesterol (TC) levels compared with NC (TG; NC 10.1 ± 2.9 mg/g liver, HFD 340.5 ± 3.6, *p* < 0.0001, TC; NC 2.8 ± 0.2 mg/g liver, HFD 26.6 ± 0.8, *p* < 0.0001, Fig. 1b). C3d deposition, a sensitive indicator of complement activation, was used to evaluate HFD-induced complement activation in the liver^18^. C3d deposition was significantly increased in HFD-fed mice compared with NC (NC 6.094 ± 0.443 mm^2^/10^5^ hepatocytes, HFD 8.448 ± 0.473, *p* < 0.01, Fig. 1c), suggesting an initiation of complement activation and subsequent inflammation in the liver following 17 weeks of HFD. To further characterize the mechanism of complement activation, mRNA expression levels of various complement components were measured in HFD-fed mice. As shown in Fig. 1d, the expression levels of *C1q*, *C3*, *C4B*, *Factor B*, *Factor D, Masp-1*, *Masp-2*, and *DAF* were significantly higher in the HFD group. *Factor D* mRNA levels increased more than 100-fold following HFD. Concomitantly, Factor D protein levels increased in livers of HFD-fed mice (NC 1.00 ± 0.15 relative expression, HFD 3.26 ± 0.76, *p* < 0.05, Fig. 1e, Supplementary Fig. 1). These results indicate a crucial role for Factor D in the development of HFD-induced lipid accumulation and subsequent inflammation in the liver.

### Generation of Factor D deficient mice with the CRISPR/Cas9 system

**Figure 2:**
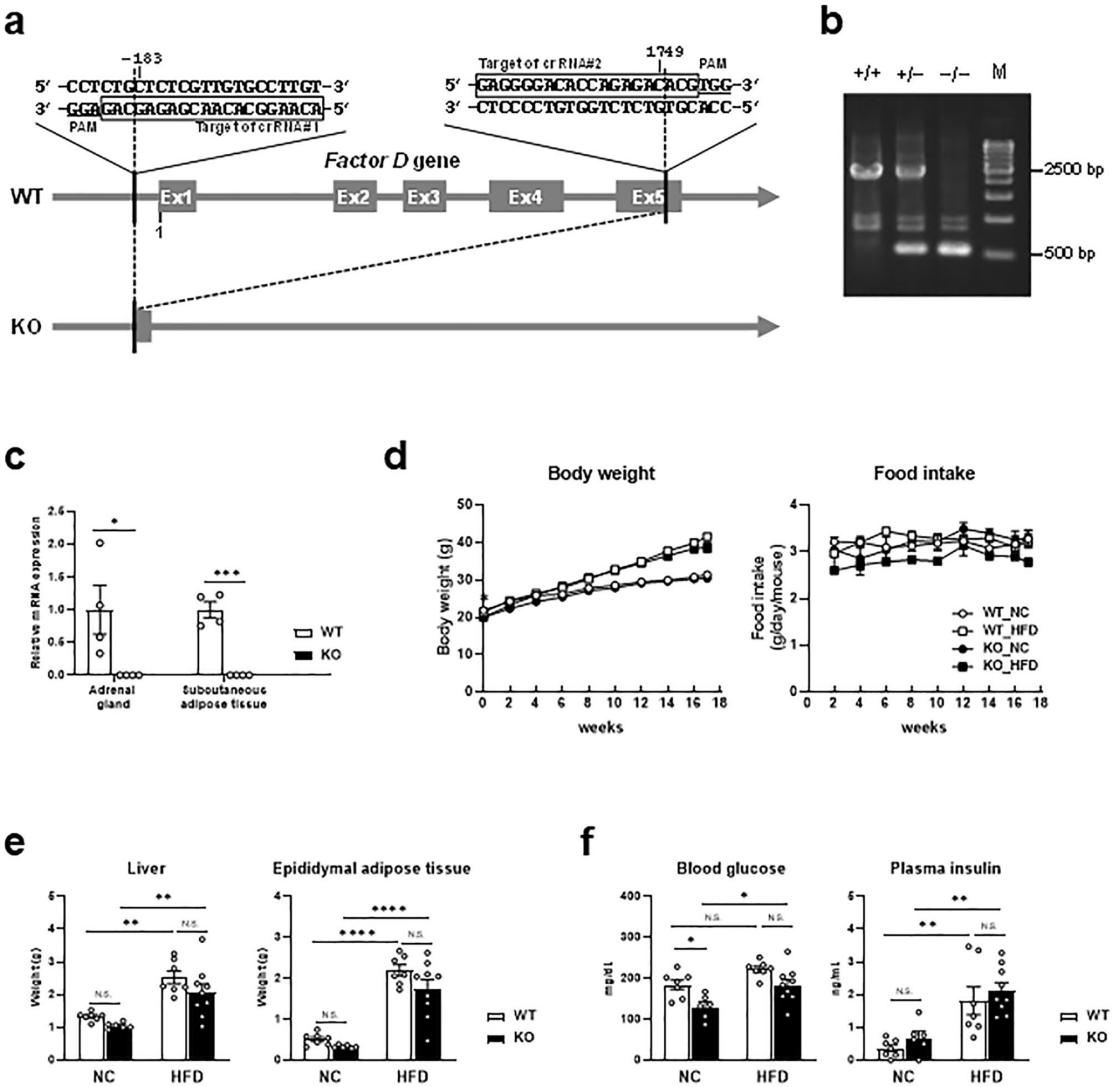
Generation of FD-KO mice. **(a**) Scheme of gene editing for deficiency of *Factor D* gene by cloning free CRISPR/Cas9 system. Exons are showed by gray boxes and introns are indicated by gray lines. The sequences in squares are target of crRNA. PAM sequences are showed by underline. (**b)** Genotyping of FD-KO mice. 2462-bp band shows wild-type allele, and 531-bp band shows knockout allele. M shows molecular weight marker. This image was cropped and the full image was shown in Supplementary Fig. 2. (**c**) Relative mRNA expression levels of *Factor D* in the adrenal gland and subcutaneous adipose tissue of WT or FD-KO mice. N = 4 in each group. Data are shown as mean ± SEM. *p < 0.05, ***p < 0.001 by an unpaired two-tailed *t* test. (**d**) Body weight and food intake, (**e**) weight of liver or epididymal adipose tissue, (**f)** glucose and insulin level in blood of WT or FD-KO mice. N = 7, 6, 7, 9. *p < 0.05, ** *p* < 0.01, ****p < 0.0001 by one-way ANOVA followed by a Tukey's *post-hoc* test.

To demonstrate a pathophysiological role for Factor D in HFD-induced hepatic steatosis, we generated Factor D knockout (FD-KO) mice using the CRISPR/Cas9 system as described in Methods (Fig. 2a). *Factor D* gene knockout was confirmed based on a disappearance of PCR products (Fig. 2b, Supplementary Fig. 2) and by the absence of *Factor D* mRNA in adrenal gland and subcutaneous adipose tissue (Fig. 2c)—sites of abundant Factor D expression in WT mice. Body weight and food intake of FD-KO mice were not significantly different from WT mice fed HFD or NC (Fig. 2d). Liver weight, epididymal adipose tissue weight (Fig. 2e), plasma glucose and insulin levels were not significantly different between FD-KO mice and WT mice fed HFD (Fig. 2f).

### Factor D deficiency decreased HFD-induced hepatic lipid accumulation in mice

**Figure 3:**
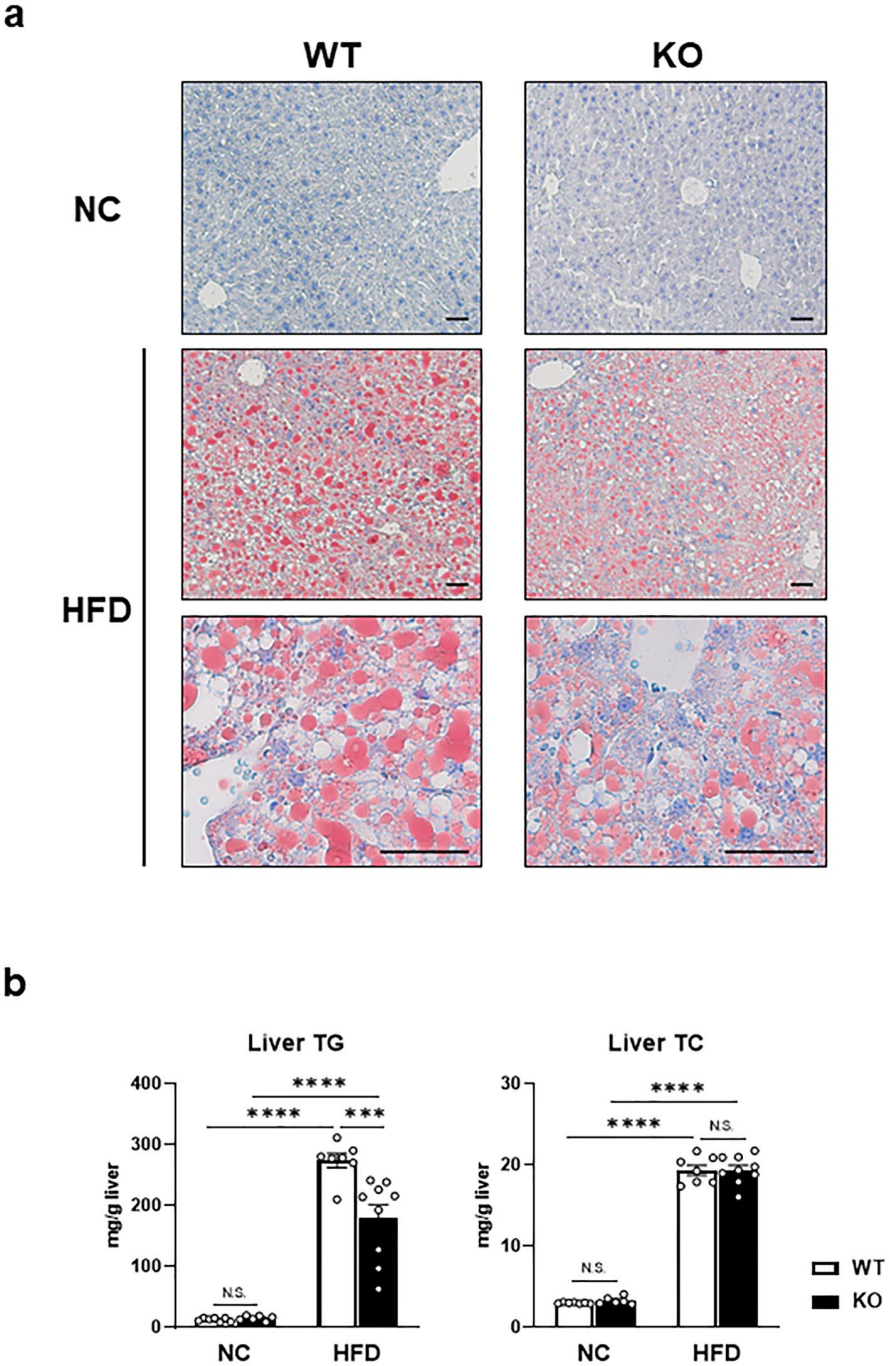
HFD-induced hepatic lipid accumulation was attenuated in FD-KO mice. **(a)** Images of Oil Red O staining for liver sections of WT mice or FD-KO mice fed NC or HFD for 17 weeks. Bars, 50 μm. The magnification of objective was  ×10 in upper and middle images, and was  ×40 in lower images. (**b**) TG or TC contents in liver of WT mice or FD-KO mice fed NC or HFD for 17 weeks. N = 7, 6, 7, 9. ***p < 0.001, ****p < 0.0001 by one-way ANOVA followed by a Tukey's post-hoc test.

To investigate the role of Factor D in hepatic lipid accumulation, Oil Red O-stained liver sections of FD-KO and WT mice were examined from HFD-fed and NC-fed mice. As shown in Fig. 3a, significant hepatic lipid accumulation was observed in WT mice fed a HFD. In contrast, FD-KO mice fed HFD, showed a clearly reduced level of lipid accumulation. Additionally, hepatic TG content was increased in WT mice after HFD whereas TG content was significantly decreased in FD-KO mice on the HFD (WT_HFD 274.1 ± 11.8 mg/g liver, KO_HFD, 179.6 ± 22.1, *p* < 0.001, Fig. 3b). In contrast, hepatic TC content was no different between FD-KO mice and WT mice after HFD (WT_HFD 19.3 ± 0.6 mg/g, KO_HFD 19.4 ± 0.6, Fig. 3b). These data suggest that Factor D involved in lipid accumulation in the liver.

### Factor D deficiency attenuated HFD-induced hepatic inflammation and fibrosis

**Figure 4:**
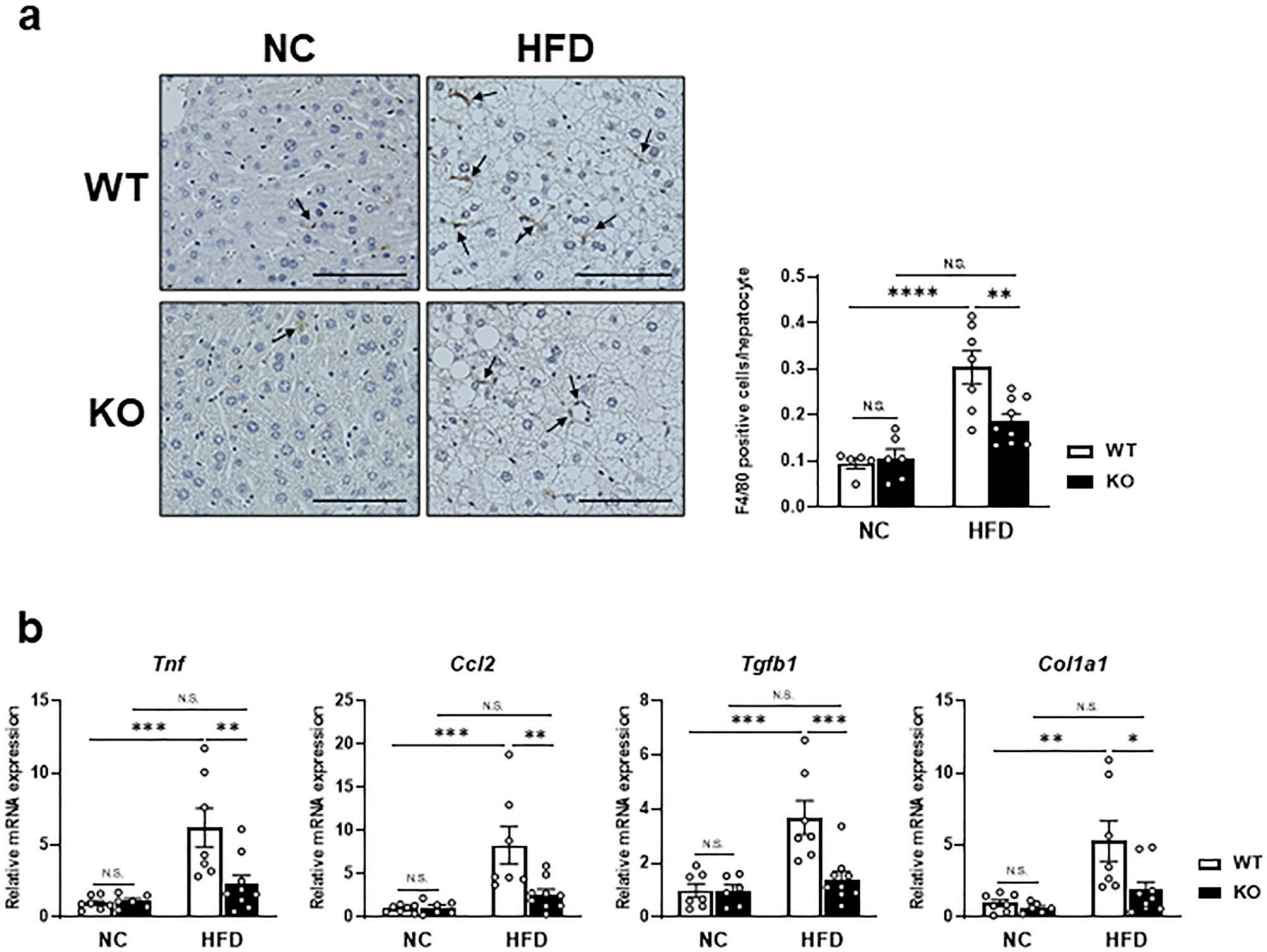
The accumulation of F4/80-positive macrophages in liver increased by HFD decreased in FD-KO mice.** (a**) Images of immunohistochemistry for F4/80 in WT mice or FD-KO mice fed with NC or HFD for 17 weeks (left panels). Black arrows indicate F4/80-positive cells. Bars, 100 μm. Magnification of objective is  × 20. Right graph shows quantification of F4/80-positive cells. N = 5, 6, 7, 9. (**b**) Relative mRNA expression levels of *Tnf*, *Ccl2*, *Tgfb1*, and *Col1a1* in liver of WT mice or FD-KO mice fed NC or HFD for 17 weeks. N = 7, 6, 7, 9. *p < 0.05, **p < 0.01, ***p < 0.001, **** p < 0.0001 by one-way ANOVA followed by a Tukey's post-hoc test.

Because Factor D and complement activation play an important role in initiating the inflammatory cascade, we examined the influence of Factor D on HFD-induced hepatic inflammation and fibrosis. Immuno-staining of F4/80, a macrophage lineage marker, revealed an increase of F4/80-positive cells in the liver of WT mice after HFD. Although FD-KO mice fed a HFD also exhibited increased F4/80 staining compared with KO mice fed a NC, increased staining with HFD was significantly less than in WT mice (WT_NC 0.09 ± 0.01 cells/hepatocyte, WT_HFD 0.30 ± 0.04, KO_NC 0.11 ± 0.02, KO_HFD, 0.19 ± 0.02, WT_NC vs WT_HFD *p* < 0.0001, WT_HFD vs KO_HFD *p* < 0.01, Fig. 4a). In correlation with macrophage results, a significant reduction in inflammatory molecules, including *Tnf, Ccl2*, *Tgfb1* and *Col1a1*, was observed in HFD-fed FD-KO mice compared with HFD-fed WT mice (Fig. 4b). These results suggest a potential role for Factor D in the inflammatory cascade in liver.

### Factor D deficiency decreased mRNA levels of genes related to de novo lipogenesis and fatty acid uptake in liver

**Figure 5:**
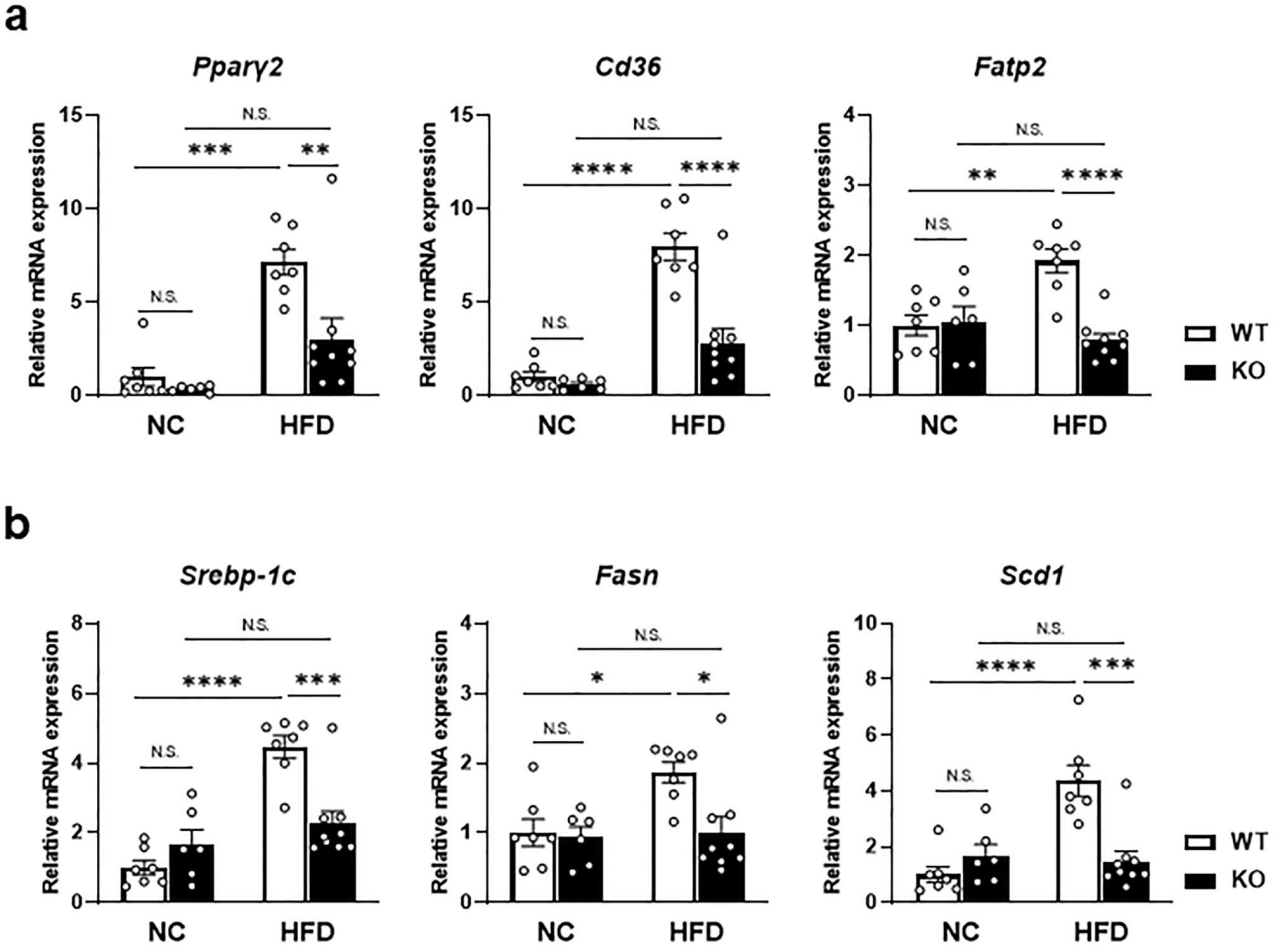
mRNA expression levels of the lipid metabolism-related genes increased by HFD were decreased in FD-KO mice. **(a**) Relative mRNA expression levels of the genes related to fatty acid uptake (*Pparγ2*, *Cd36*, and *Fatp2*), and (**b)** de novo lipogenesis (*Srebp-1c*, *Fasn*, and *Scd1*) in liver of WT mice or FD-KO mice fed with NC or HFD for 17 weeks. N = 7, 6, 7, 9. *p < 0.05, **p < 0.01, ***p < 0.001, **** *p* < 0.0001 by one-way ANOVA followed by a Tukey's *post-hoc* test.

To further explore the mechanism by which Factor D regulates lipid accumulation after HFD, expression levels of fatty acid uptake and de novo fatty acid synthesis factors were examined. As shown in Fig. 5a, mRNA levels of genes related to fatty acid uptake (*Pparγ2*, *Cd36* and *Fatp2*) were significantly less in FD-KO mice fed HFD compared with WT mice fed HFD (Fig. 5a). Similarly, mRNA levels of genes related to de novo lipogenesis (*Srebp-1c*, *Fasn* and *Scd1*) were also significantly reduced in HFD-fed FD-KO mice compared with HFD-fed WT mice (Fig. 5b). These results indicate that HFD-induced lipid accumulation in the liver is modulated by Factor D’s effects on both fatty acid uptake and de novo lipogenesis.

## Discussion

In this study, we identified a pivotal role for Factor D in the development of HFD-induced NAFLD in mice. Mechanistically, Factor D acts via enhancing uptake and de novo synthesis of fatty acids in the liver. These findings suggest a link between complement activation and lipid accumulation in the liver via Factor D-dependent pathways.

NAFLD has been regarded as a hepatic manifestation of metabolic syndrome^3^. NAFLD is characterized by excessive accumulation of TG in the liver, visceral adipose tissue accumulation and insulin resistance with elevated levels of circulating free fatty acids^19,20^. Various inflammatory cytokines within the hepatic tissue as well as microbial derivatives from intestine also play a role in the process^21,22^.

Although previous studies have shown that the complement system is important in the development of NAFLD, a critical role for Factor D has not been previously studied. We found that hepatic lipid accumulation in WT mice fed HFD was significantly reduced in FD-KO mice fed the same diet (Fig. 3). FD-KO mice exhibited diminished infiltration of F4/80-positive macrophages and reduced expression of inflammatory cytokines in the liver (Fig. 4). The enhanced inflammatory response by HFD induced the expression of SRBP-1c and SCD1^23^. Factor D deficiency may diminish the expression of SREBP-1c and SCD1 through the attenuation of inflammation. The magnitude of Factor D elevation in the liver of WT mice fed HFD (Fig. 1) was significantly higher than elevations of other complement system components, indicating that Factor D plays an important role in accumulation of TG and the subsequent initiation of inflammatory cascades in the liver. Previous studies have reported critical roles for Factor D in pancreatic β cell insulin stimulation and adipocyte TG synthesis^15,16^, supporting the findings of the current study. These data implicate Factor D in various metabolic tissues, including β cells, adipocytes and hepatocytes. Interestingly, however, we failed to detect blood glucose elevation in FD-KO mice fed HFD for up to 17 weeks. Thus, Factor-D-dependent hepatic lipid accumulation appear independent from subsequent insulin resistance observed after 17 weeks of HFD.

Previous studies have established that C1q or C3a receptor deficiency reduced hepatic lipid accumulation in mice fed HFD^24,25^. In contrast, triglyceride levels did not change in livers of C3 null mice^26^. Therefore, a causative role for the complement pathway in the development of NAFLD remains unclear. Moreover, FD-KO mice are susceptible to bacterial infections due to the disruption of inflammatory signaling pathway^27^. Excess hepatic lipid accumulation as an inflammatory stimulus may also cause similar disruption of inflammatory signaling that is manifested as a reduction in inflammatory markers as shown Fig. 4.Further investigation is needed to determine whether Factor D acts via a novel mechanism independent of the complement pathway to stimulate hepatic lipid accumulation.

Factor D impacted key elements in both fatty acid uptake and de novo lipogenesis (Fig. 5). FD-KO mice fed HFD exhibited reduced levels of peroxisome proliferator activated receptor gamma (PPARγ), which has been shown to play an important role in lipid metabolism in liver as well as other metabolic organs such as adipose tissues and macrophages^28,29^. Additionally, FD-KO mice had reduced expression of fatty acid transporters such as *Cd36* and *FATP2* (fatty acid transport protein 2)^30,31^. These lines of evidences indicate that Factor D may activate PPARγ/CD36/FATP2 axis to enhance hepatic lipid uptake upon high-fat diet. Moreover, FD-KO mice had reduced expression of SREBP-1c, a molecule known to enhance transcription of lipogenic genes including fatty acid synthase (FASN) and stearoyl-CoA desaturase 1 (SCD1)^32^. These data implicate that de novo fatty acid synthesis is also affected.

**Figure 6:**
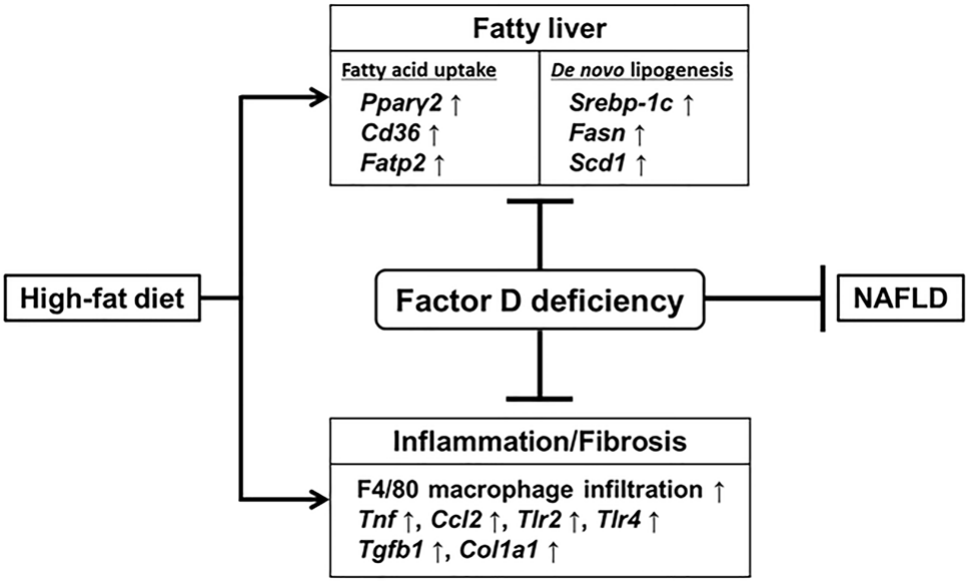
Scheme of the mechanism of suppression of hepatic lipid accumulation and inflammation by Factor D deficiency in mouse. Deficiency of Factor D increased by HFD-feeding reduced hepatic lipid accumulation and inflammatory factors in the liver.

In conclusion, the current study indicated that Factor D may be a crucial component in HFD-induced hepatic lipid accumulation and hepatic inflammation (Fig. 6). These data suggest that Factor D has potential for involvement in the development of NAFLD.

## Materials and methods

### Generation of Factor D knockout mice by the CRISPR/Cas9 system

To investigate the involvement of Factor D in hepatic lipid accumulation, we generated Factor D knockout (FD-KO) mice using the CRISPR/Cas9 genome-editing system as previously described^33^. To diminish the function of Factor D entirely, the region from exon1 to exon5 was deleted using two target sequences, crRNAs directed to the 5′ side in exon1 and the 3′ side in exon5 (Fig. 2a). Sequences of each crRNA and tracrRNA are described in Supplementary Table 1. A single-stranded oligo DNA donor (Supplementary Table 1), which contains 75 bp up- or downstream region from DNA double-strand breaking sites determined by each crRNAs, were coinjected to a fertilized egg with all the CRISPR/Cas9 components including crRNAs and a trans-activating crRNA.. A mixture of Cas9 protein, Factor D crRNA and tracrRNA was injected into single cell zygotes obtained from C57BL/6J mice. Following maturation to the two-cell stage, embryos were transferred to female mice. New-borns were screened by polymerase chain reaction (PCR), and founder mice were crossed with C57BL/6J mice. PCR with primer set #1 and #2 (Supplementary Table 1) was used to determine the genotypes of offspring. Additional PCR using primer set #3 and #4 (Supplementary Table 1) was used to confirm genotype. Homozygous Factor D deficient mice (FD-KO) were selected for this study (Fig. 2b). Using quantitative RT-PCR, diminished Factor D mRNA expression in adrenal gland or subcutaneous adipose tissue was used to confirm knockout status (Fig. 2c).

Seven-week-old male wild type C57BL/6J (WT) mice were obtained from Charles River Laboratories Japan, Inc. WT mice or FD-KO mice were fed normal chow (NC, CE-2, Clea Japan, Inc.) or HFD (1.25% cholesterol, 20% tallow in F-2, Sankyo Labo Service Corporation, Inc.) for 17 weeks. Food and water were provided ad libitum. Mice were either fasted for 5 h or not fasted prior to experiments. Experiments adhered to the APS Guiding Principles on the Care and Use of Animals and were approved by the Ethical Committee for Animal Experimentation of Tokyo Medical and Dental University.

### RT-quantitative PCR

Mice were anesthetized with pentobarbital and livers were harvested. Total RNA was isolated from approximately 100 mg of liver using TRIzol Reagent (Thermo Fisher Scientific Inc.). Quantitative RT-qPCR was performed on the Thermal Cycler Dice Real Time System TP850 (Takara Bio Inc.) using specific primers (Supplementary Table 2) as previously reported^34^. Relative mRNA expression levels were calculated using the standard curve method and normalized to 18S ribosomal RNA internal control.

### TC and TG levels in liver

Liver lipids were extracted as previously described and 100 mg of lipids were dissolved in chloroform–methanol (2:1, v/v)^35,36^. Total cholesterol or triglyceride in liver were measured using LabAssay Cholesterol (Fujifilm Wako Pure Chemical Corporation) or LabAssay Triglyceride (Fujifilm Wako Pure Chemical Corporation).

### Blood glucose and insulin levels

Glutest Ace R or Glutest ai (Sanwa Kagaku Kenkyusho Co. Ltd.) was used to measure glucose levels in tail vein blood according to manufacturer’s protocol.

Blood obtained via cardiac puncture was collected in EDTA tubes, and plasma was harvested. Plasma insulin levels were determined using a commercial ELISA kit (Mercodia AB) according to the manufacturer’s protocol.

### Tissue histology

Liver samples were fixed overnight in 4% paraformaldehyde for cryosections, or 10% neutral buffered formalin for paraffin embedding. Endogenous biotin-blocked, 4-µm-thick paraffin sections were stained with haematoxylin, anti-C3d antibody (R&D Systems, Inc.) or anti-F4/80 antibody (Biolegend, Inc.)^37,38^. Six-µm-thick cryosections were stained with Oil Red O (Sigma-Aldrich) and Mayer's Hematoxylin Solution (Fujifilm Wako Pure Chemical Corporation)^39^. Captured images were analysed using the BZ-X800 Analyzer (Keyence Corporation) or ImageJ (Fiji)^40^. C3d-positive area and F4/80-positive cells per hepatocyte were calculated.

### Western blotting

Livers were lysed in RIPA buffer, and 10 µg of protein was used for SDS‐PAGE. Separated proteins were transferred to PVDF membrane and reacted with anti-mouse Factor D antibody (Cloud-Clone Corp.) or anti-Hsc70 polyclonal antibody (Bio Vision, Inc.) as equal loading controls. Bands were detected and analysed with the LAS1000 (Fujifilm Corp.).

### Statistical analysis

All results were expressed as mean ± standard error of the mean (SEM). Statistical differences were determined by one-way ANOVA with a post-hoc Tukey's or Dunnett's test or by 2-tailed Student’s or Welch's *t* test. *p* < 0.05 was considered significant.

## Supplementary information

Supplementary Information.
