# Supplementary material for: HFD-induced hepatic lipid accumulation and inflammation are decreased in Factor D deficient mouse
Source: Sci Rep. 2020 Oct 16;10:17593. doi: 10.1038/s41598-020-74617-5 (PMC7568538; doi:10.1038/s41598-020-74617-5)
Supplement: Supplementary file 1 — Supplementary Information. [file 41598_2020_74617_MOESM1_ESM.pdf]

Supplementary Materials for “HFD-induced hepatic lipid accumulation and inflammation are decreased in Factor D deficient mouse.”

Hiromi Tsuru[1], Mizuko Osaka[1, 2], Yuichi Hiraoka[3], Masayuki Yoshida[1]*.

[1]Department of Life Sciences and Bioethics, Graduate School of Medical and Dental Sciences, Tokyo Medical and Dental University, Tokyo, Japan

[2]Department of Nutrition and Metabolism in Cardiovascular Disease, Graduate School of Medicine and Dental Sciences, Tokyo Medical and Dental University, Tokyo, Japan

[3]Laboratory of Molecular Neuroscience, Medical Research Institute, Tokyo Medical and Dental University, Tokyo, Japan

*Corresponding author:: Masayuki Yoshida, MD

This file include Supplementary Fig1, Fig 2, Table 1 and 2.

Supplementary Fig. 1

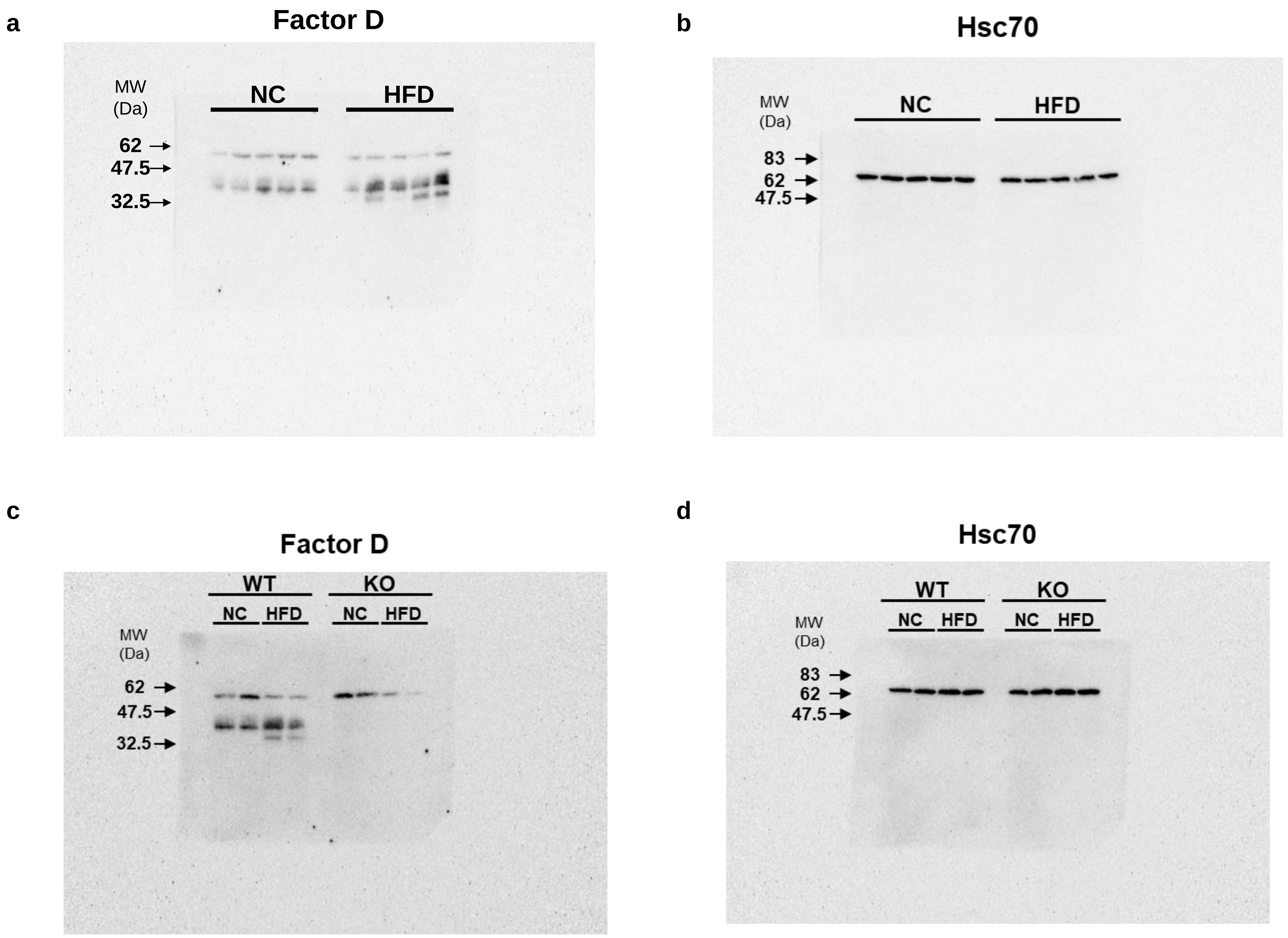

Supplementary Fig. 1 Full image of westernblotting for Factor D in WT mice fed NC or HFD.

a. Westernblotting for Factor D in WT mice fed NC or HFD.

b. Westernblotting for Hsc 70 as equal loading control for Fig 1a.

c. Westernblotting for Factor D in FD-KO mice fed NC or HFD.

d. Westernblotting for HSC 70 as equal loading control for Fig 1c.

Supplementary Fig. 2

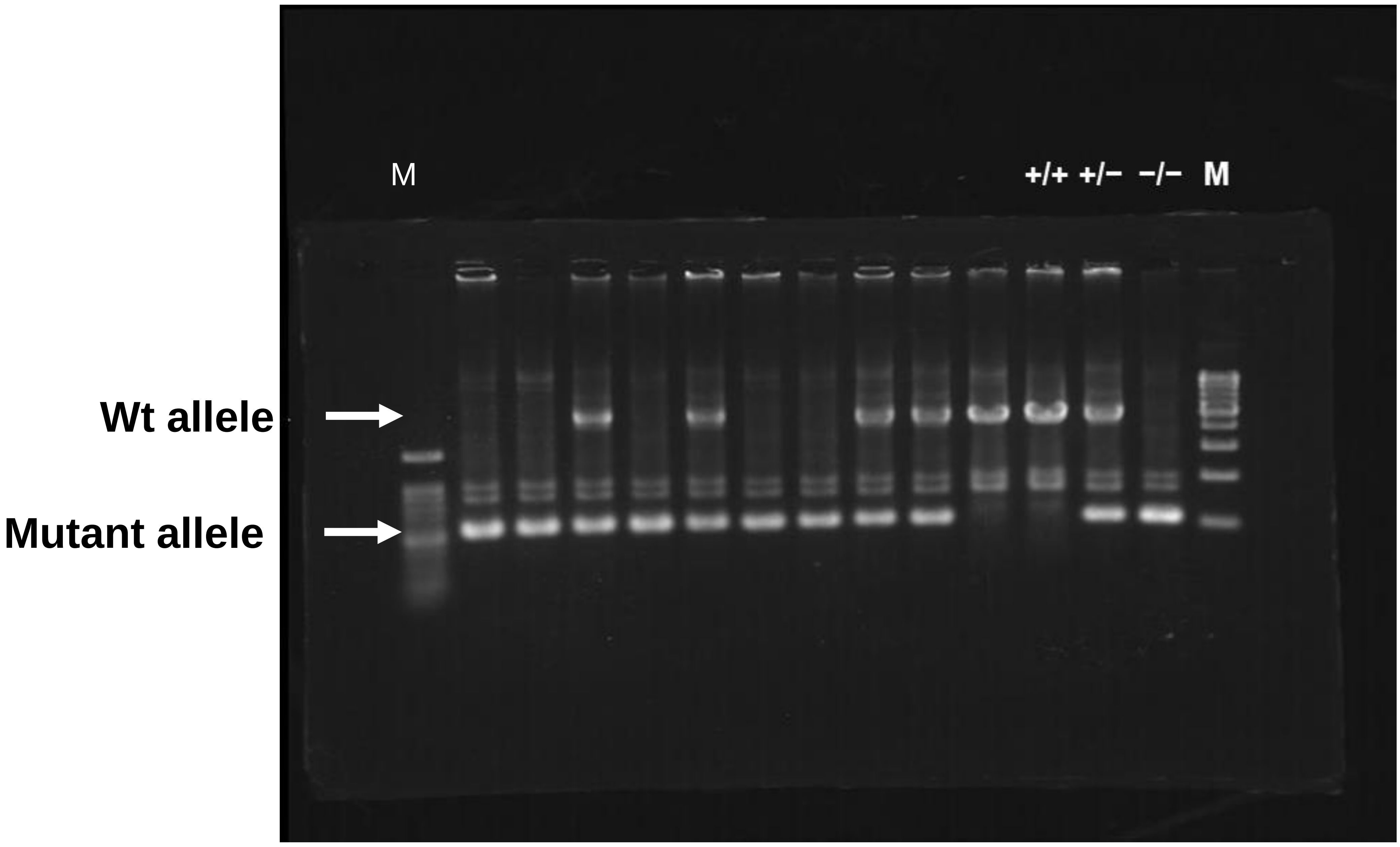

Supplementary Fig. 2 Full image of agarose gel for genotyping of FD – KO mice.

Wt allele is 2462 bp, mutant allele is 531 bp.

Supplementary Table1. Primers for generation of FD knockout mice by CRISPR-Cas9 system.

**crRNA#1**	5′-ACAAGGCACAACGAGAGCAGguuuuagagcuaugcuguuuug-3′
**crRNA#2**	5′-GAGGGGACACCAGAGACACGguuuuagagcuaugcuguuuug-3′
**tracrRNA**	5′-AAACAGCAUAGCAAGUUAAAAUAAGGCUAGUCCGUUAUCAACUUGAAAAAGUGGCACCGAGUCGGUGCU-3′
**Oligo DNA donor**	5′-TTTAACAGACACCATGACAGACACATGGCTGGAAGGATACTCACTGGGCCAGGACAACAGGTGGTCCATCCTCTGACGTGGCTCAGGGAAACAAGAGACACGTGGCTCACAATAAATGCATGCATCTGAGCCCTGTGCATCTTTCTTTTT-3′
**Primer#1**	5′-ATGGGGTGGAGGGTGTTACT-3′
**Primer#2**	5′-ACAACAGTCCTGGGTACAGC-3′
**Primer#3**	5′-GCCGACCTGACAGCCTTGAG-3′
**Primer#4**	5′-TTCCACTTCTTTGTCCTCGTATTGC-3′

FD specific sequences in crRNA are underlined.

Supplementary Table 2. Primers for RT-qPCR.

Gene	Forward primer	Reverse primer
Factor D	5'-GCCGACCTGACAGCCTTGAG-3'	5'-TTCCACTTCTTTGTCCTCGTATTGC-3'
C1q	5'-CTCTTTCAGGTGTTAGCAGG-3'	5'-AAGATGCTGTCGGCTTCAGT-3'
C2	5'-TGCCAGCTACAAAGATCACG-3'	5'-ATTTCCTTCCAGGCAGAGGT-3'
C3	5'-GACGCCACTATGTCCATCCT-3'	5'-TACTCCAGAGGCCAGCAGTT-3'
C4A	5'-TCAGGCTCTGAAGCCTCTAA-3'	5'-CTCTGTTGAACGTTGAGGAC-3'
C4B	5'-AGAGCACCAATGGAATTGGC-3'	5'-GAGTCGAAGTTCTGTGACAG-3'
Factor B	5'-CTCCTCTGGAGGTGTGAGCG-3'	5'-GGTCGTGGGCAGCGTATTG-3'
C5	5'-TGAGCGTCATGTCCTACAGA-3'	5'-CACCTGTCCAAGCACTCTCA-3'
Masp-1	5'-AGGACCTGCCGAGTGGAATG-3'	5'-TCTCCACAGAAGGGACCCCA-3'
Masp-2	5'-CAGCGGAGGATGTGGATGAATG-3'	5'-GTTCTGGTGGAGAACGTAGCC-3'
DAF	5'-TGTAAGCAGAATCGCCACAG-3'	5'-AGCTTCCACTGCAGGTTTGT-3'
Pparγ2	5'-TTCGCTGATGCACTGCCTAT-3'	5'-GGAATGCGAGTGGTCTTCCA-3'
Cd36	5'-GGAGCCATCTTTGAGCCTTC-3'	5'-TGGATCTTTGTAACCCCACAAG-3'
Fatp2	5'-GCTGACATCGTGGGACTGGT-3'	5'-TTCGACCCTCATGACCTGGC-3'
Srebp-1c	5'-GGAGCCATGGATTGCACATT-3'	5'-GGCCCGGGAAGTCACTGT-3'
Fasn	5'-TGAGCACACTGCTGGTGAAC-3'	5'-CAGGTTCGGAATGCTATCCA-3'
Scd1	5'-ATCGCCCCTACGACAAGAAC-3'	5'-GTTGATGTGCCAGCGGTACT-3'
Tnf	5'-GCCTCTTCTCATTCCTGCTTG-3'	5'-CTGATGAGAGGGAGGCCATT-3'
Ccl2	5'-CTGTGCTCAGAGCTTTCAAC-3'	5'-TCTCCCTTTGCAGAACTCAG-3'
Tgfb1	5'-CGCCATCTATGAGAAAACC-3'	5'-GTAACGCCAGGAATTGT-3'
Col1a1	5'-ATGTTCAGCTTTGTGGACCTC-3'	5'-CAGAAAGCACAGCACTCGC-3'
18S RNA	5'-GTAACCCGTTGAACCCCATT-3'	5'-CCATCCAATCGGTAGTAGCG-3'
